# Supplementary material for: Generation of mature compact ventricular cardiomyocytes from human pluripotent stem cells
Source: Nat Commun. 2021 May 26;12:3155. doi: 10.1038/s41467-021-23329-z (PMC8155185; doi:10.1038/s41467-021-23329-z)
Supplement: Supplementary file 1 — Supplementary Information [file 41467_2021_23329_MOESM1_ESM.pdf]

Supplementary information

Generation of mature compact ventricular cardiomyocytes from human pluripotent stem cells

Shunsuke Funakoshi et al.

Supplementary Figure 1

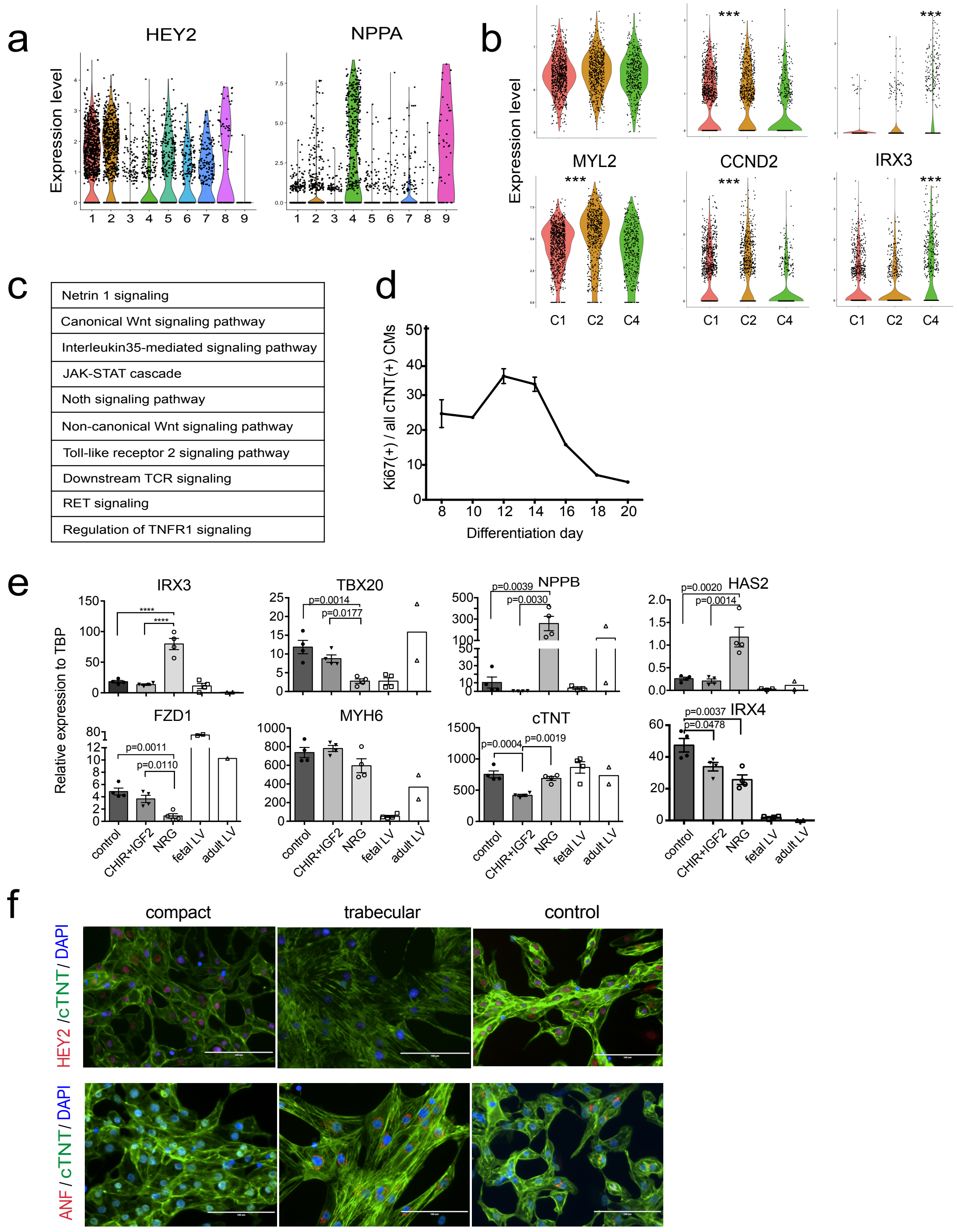

Supplementary Figure 1. Generation of compact cardiomyocytes. (a) Violin plots of the expression patterns of *HEY2* and *NPPA* in the different clusters. (b) Violin plots of the indicated genes in the two *HEY2*^{high} clusters (cluster 1 and 2, pink and yellow) and in the *NPPA*^{high} cluster

(cluster 4, green). ***p<0.001 by Wilcoxon test. (c) List of the top 10 highly upregulated signaling pathways in *HEY2*^{high} clusters compared to in *NPPA*^{high} cluster. (d) Quantification of the proportion of Ki67^{+} cardiomyocytes in the differentiating populations at the indicated times (N=4 biologically independent experiments at day 8-16, N=3 at day18-20). (e) RT-qPCR expression analyses of general cardiac markers (*cTNT*, MYH6, *IRX4*) and differentially expressed genes in the compact (CHIR+IGF2 treated), trabecular (NRG treated), and control (DMSO treated) day 16 populations (N=4). Fetal LV and RA tissues were included as a reference for *in vivo* expression. Statistical analyses were performed by one-way ANOVA with Tukey's multiple comparisons (****p<0.0001). (f) Representative immunostaining showing the proportion of HEY2^{+}cTNT^{+} and ANF^{+}cTNT^{+} cardiomyocytes in the compact, trabecular), and control day 16 populations. Scale bar:100um. Three independent experiments were repeated with similar results. Data are presented as mean values +/- SEM. Fetal LV: Fetal left ventricular tissue (N=2-4), Fetal RA: Fetal right atrial tissue (N=2).

Supplementary Figure 2

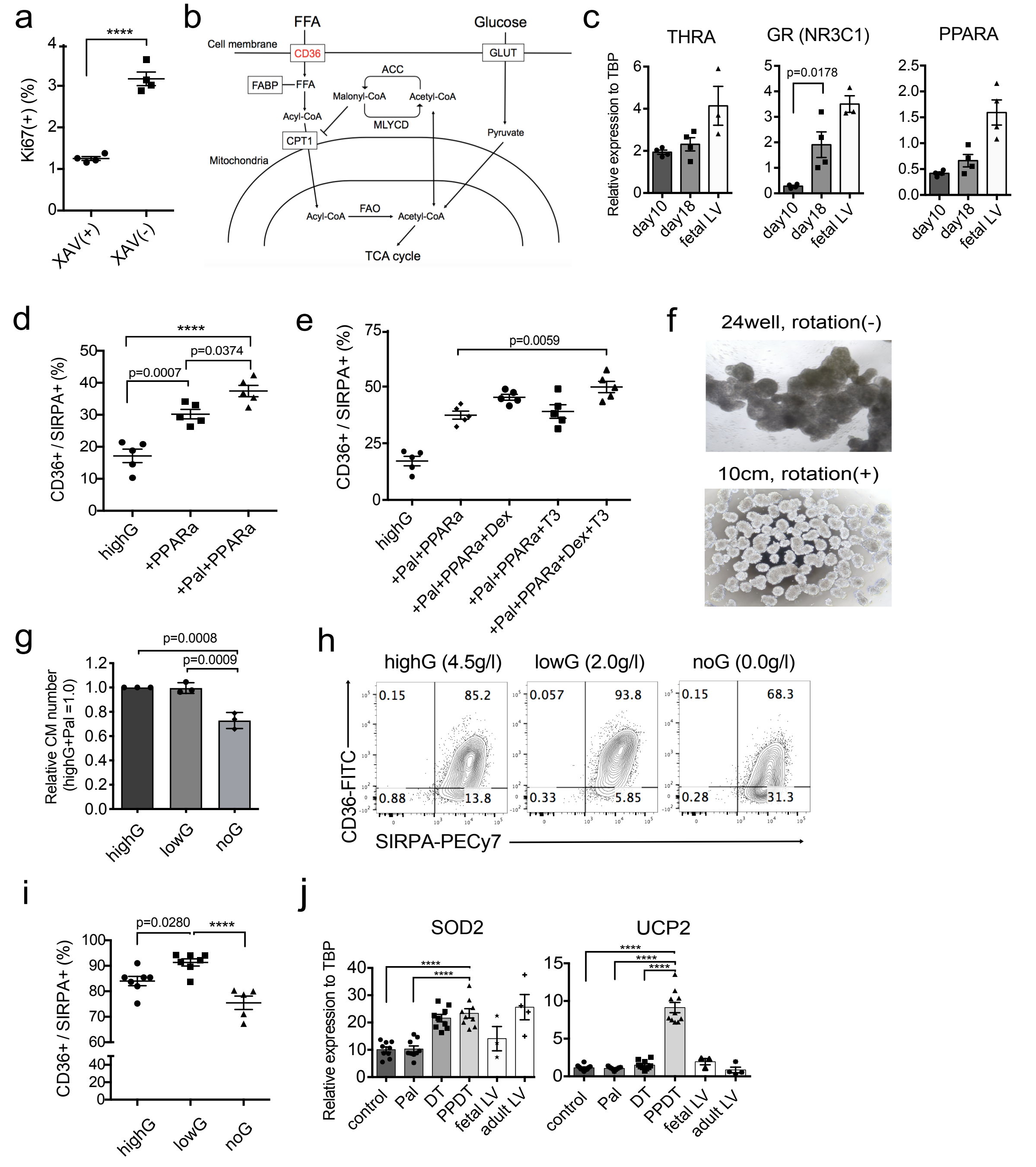

Supplementary Figure 2. Induction of FAO in compact cardiomyocytes. (a) Quantification of the percentage of Ki67$^+$ cardiomyocytes in the untreated and XAV-treated day 18 populations (N=4). (b) Schematic overview of energy metabolism in cardiomyocytes. FFA: free fatty acids. FAO: fatty acid oxidation. (c) RT-qPCR expression analyses of thyroid hormone receptor alpha

(*THRA)*, glucocorticoid receptor (*NR3C1*), and *PPARA* in day 10 and day 18 cardiomyocytes (N=4). (d, e) Quantification of the proportion of $CD36^{+}/SIRPA^{+}$ cardiomyocytes in the day 32 populations generated under the indicated conditions (N=5). (f) Photomicrograph of embryoid bodies cultured for 32 days in a 24-well culture dish without rotation (upper image) and in a 10cm dish with rotation (70rpm) (lower image). (g) Comparison of the relative number of cardiomyocytes in the day 32 populations generated in media supplemented with different concentrations of glucose (N=3). (h) Representative flow cytometric analyses of CD36 and SIRPA expression on day 32 GW/Dex/T3/Pal-treated cardiomyocytes in media containing high (4.5g/L), low (2g/L) or no glucose (0g/L). (i) Flow cytometric-based quantification of the proportion of $CD36^{+}/SIRPA^{+}$ cells in day 32 populations generated in media supplemented with different concentrations of glucose (N=7 biologically independent experiments for highG and lowG, N=5 for noG). (j) RT-qPCR expression analyses of *SOD2* and *UCP2* in the cells cultured under the indicated conditions (N=9 biologically independent samples for SOD2, N=10 samples for UCP2). Fetal LV and adult LV tissues were included as a reference for *in vivo* expression. Statistical analyses were performed by one-way ANOVA with Tukey's multiple comparisons (d-j) or two-sided unpaired t-test (a,c) (****$p<0.0001$). Data are presented as mean values +/- SEM. Fetal LV: Fetal left ventricular tissue (N=3-4), Adult LV: Adult left ventricular tissue (N=4).

Supplementary Figure 3

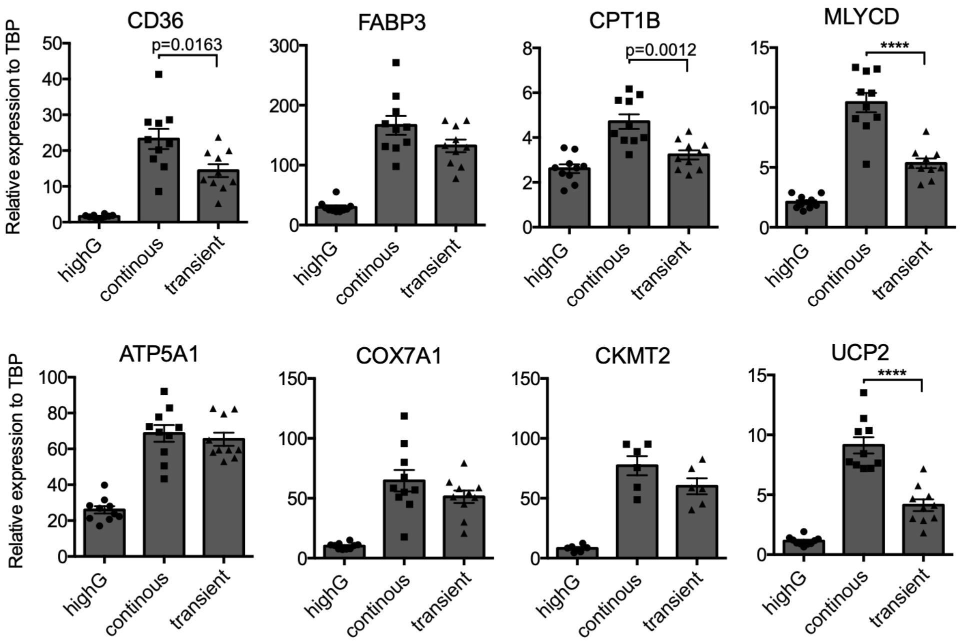

Supplementary Figure 3. Transient activation of the FAO pathway improves metabolic profiles in mature compact cardiomyocytes. RT-qPCR expression analyses of FAO- and mitochondria-related genes in control cardiomyocytes, in cardiomyocytes specified for 14 days with PPDT (continuous) and in cardiomyocytes specified for 9 days with PPDT followed by 5 days culture in Pal (transient) (N=10 biologically independent samples from each condition for CD36, FABP3, CPT1B, MLYCD, ATP5A1, COX7A1, UCP2. N=6 samples from each condition for CKMT2). Statistical analyses were performed by two-sided unpaired t-test (****$p<0.0001$). Data are presented as mean values +/- SEM.

Supplementary Figure 4

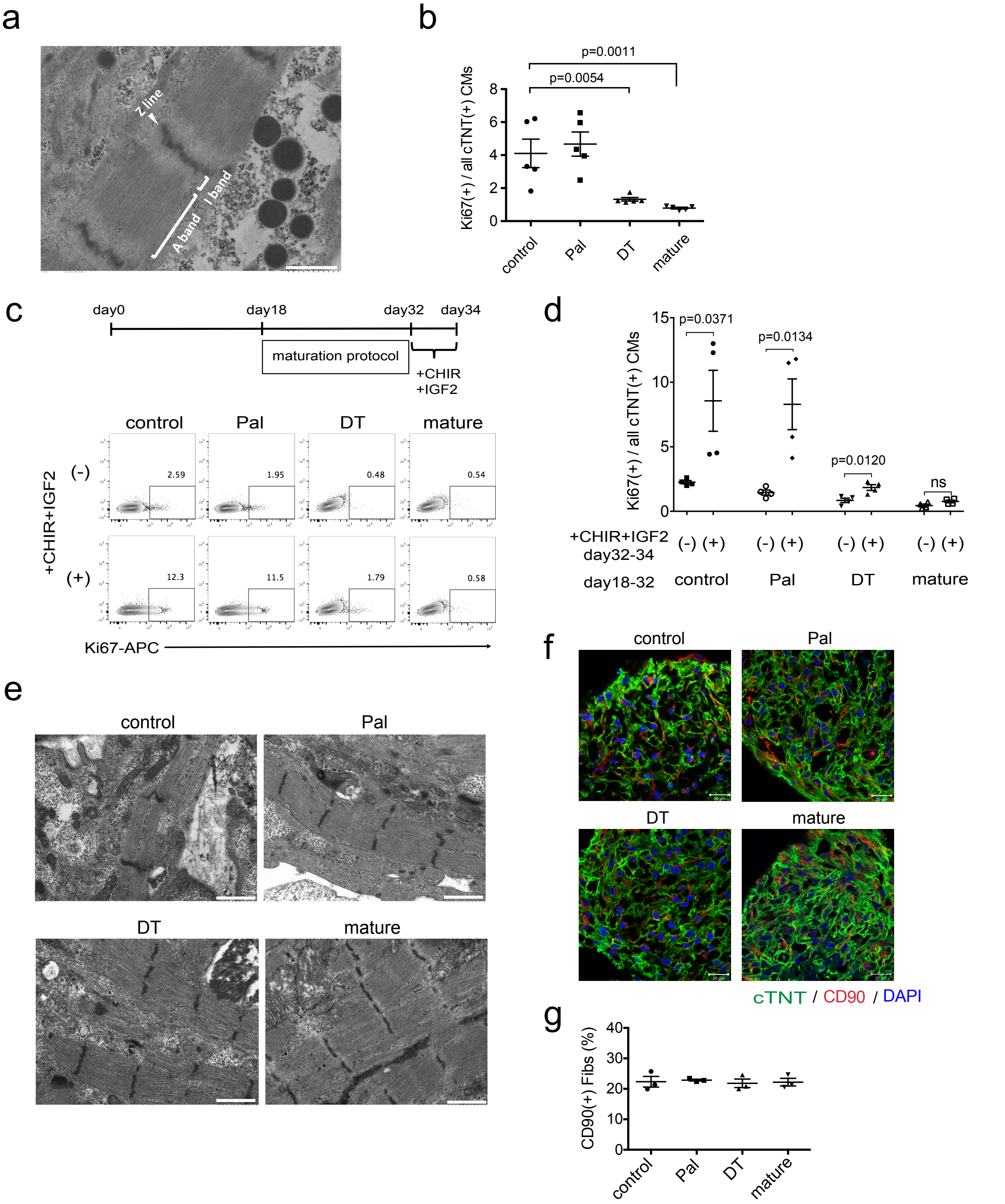

Supplementary Figure 4. Structural characteristics of metabolically mature compact cardiomyocytes. (a) Representative TEM image showing sarcomere structures including the Z line, A band, and I band in day 32 mature cardiomyocytes. Scale bar;1um. (b) Flow cytometric

quantification of the proportion of Ki67$^+$ cells in the day 32 cTNT$^+$ population generated under the indicated conditions (N=5). Statistical analyses were performed by one-way ANOVA with Tukey's multiple comparisons. (c) Upper; Schematic of protocol used to assess the responsiveness of the different populations to CHIR and IGF2. Lower; Representative flow cytometric analyses showing the proportion of Ki67$^+$ cells in day 34 cTNT$^+$ populations generated under the indicated conditions. (d) Quantification of the proportion of Ki67$^+$ cardiomyocytes in the cTNT$^+$ populations generated under the indicated conditions (N=4). Statistical analyses were performed by two-sided unpaired t-test. (e) Representative TEM images showing sarcomere structure in cells in the biowire tissues generated under the indicated conditions. Scale bar;1um. (f) Representative immunostaining showing the proportion of cTNT$^+$ and CD90$^+$ cells in the biowire tissue generated under the indicated conditions. Scale bar:20um. (g) Comparison of the percentatge of CD90$^+$ cardiac fibroblasts in biowire cardiac tissues generated under the indicated conditions (N=3). Data are presented as mean values +/- SEM.

Supplementary Figure 5

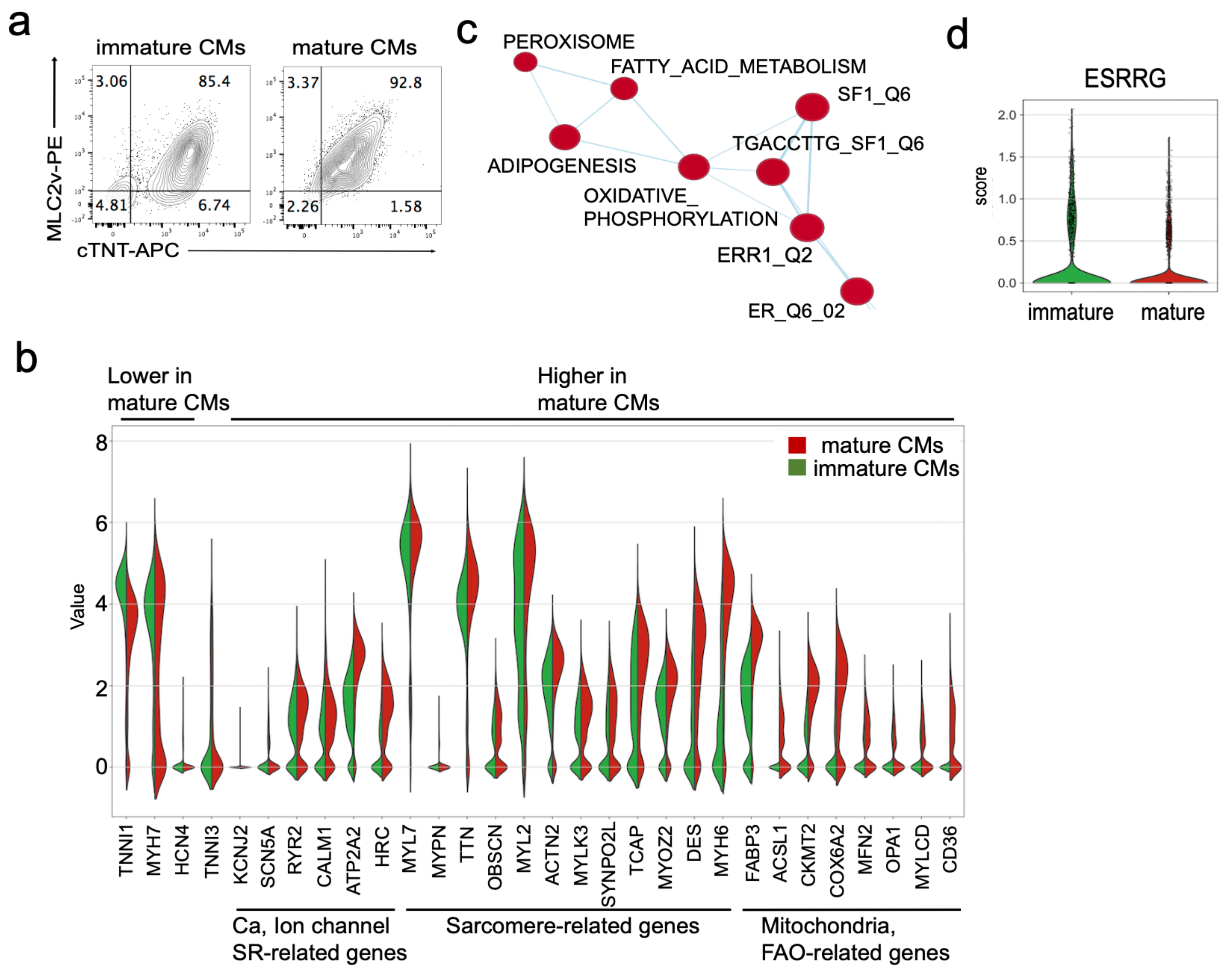

Supplementary Figure 5. Single cell RNA sequencing analysis of mature compact cardiomyocytes. (a) Representative flow cytometric analyses of MLC2v and cTNT expression in immature and mature day32 cardiomyocytes. (b) Violin plot of representative Ca^{2+}, ion channel, sarcoplasmic reticulum (SR), sarcomere, mitochondria, and FAO-related gene expression in the immature and mature cardiomyocytes. (c) Cytoscape Enrichment Map visualization of a subset of identified, enriched gene sets. (d) Violin plot of *ESRRG* expression in the immature (cluster 1, green) and mature (cluster 0, red) cardiomyocytes.

Supplementary Figure 6

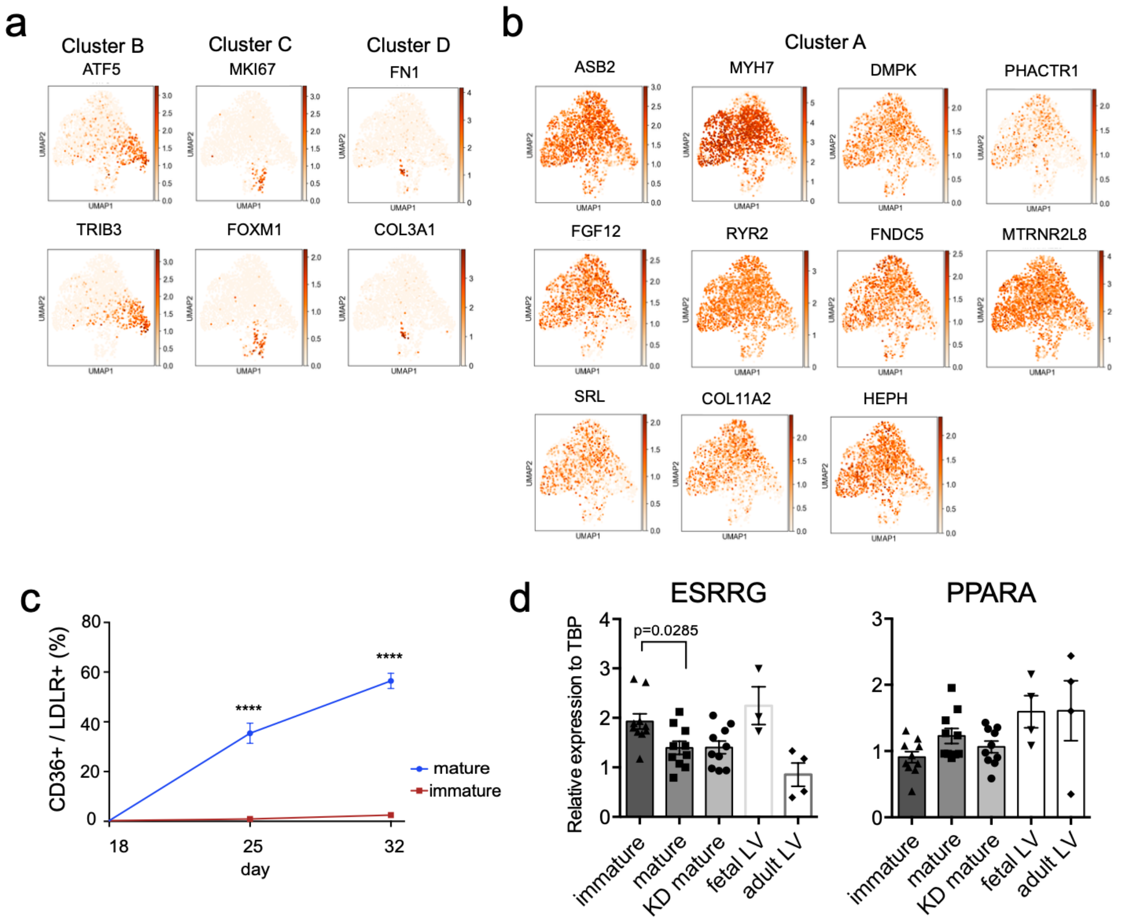

Supplementary Figure 6. Detailed molecular analyses of the mature cardiomyocyte population. (a) UMAP plots displaying expression of indicated genes in cluster B, C, and D. (b) UMAP plots displaying expression of indicated genes in cluster A. (c) Quantification of the proportion of $CD36^+/LDLR^+$ cardiomyocytes in mature and immature populations on the indicated days (mature: N=5 biologically independent experiments at day 25, N=6 at day 32, immature: N=6 at day 25, N=7 at day 32). The proportion was compared at each time point by two-sided unpaired t-test (****p<0.0001). (d) RT-qPCR expression analyses of *ESRRG* and *PPARA* in the immature, mature, and mature cardiomyocytes with ESRRA knock down (KD mature) (N=10 biologically independent experiments). Fetal LV and adult LV tissues were included as a reference for *in vivo* expression. Statistical analyses were performed by one-way ANOVA with Tukey's multiple comparisons. Data are presented as mean values +/- SEM. Fetal LV: Fetal left ventricular tissue (N=3-4), Adult LV: Adult left ventricular tissue (N=4).

Supplementary Figure 7

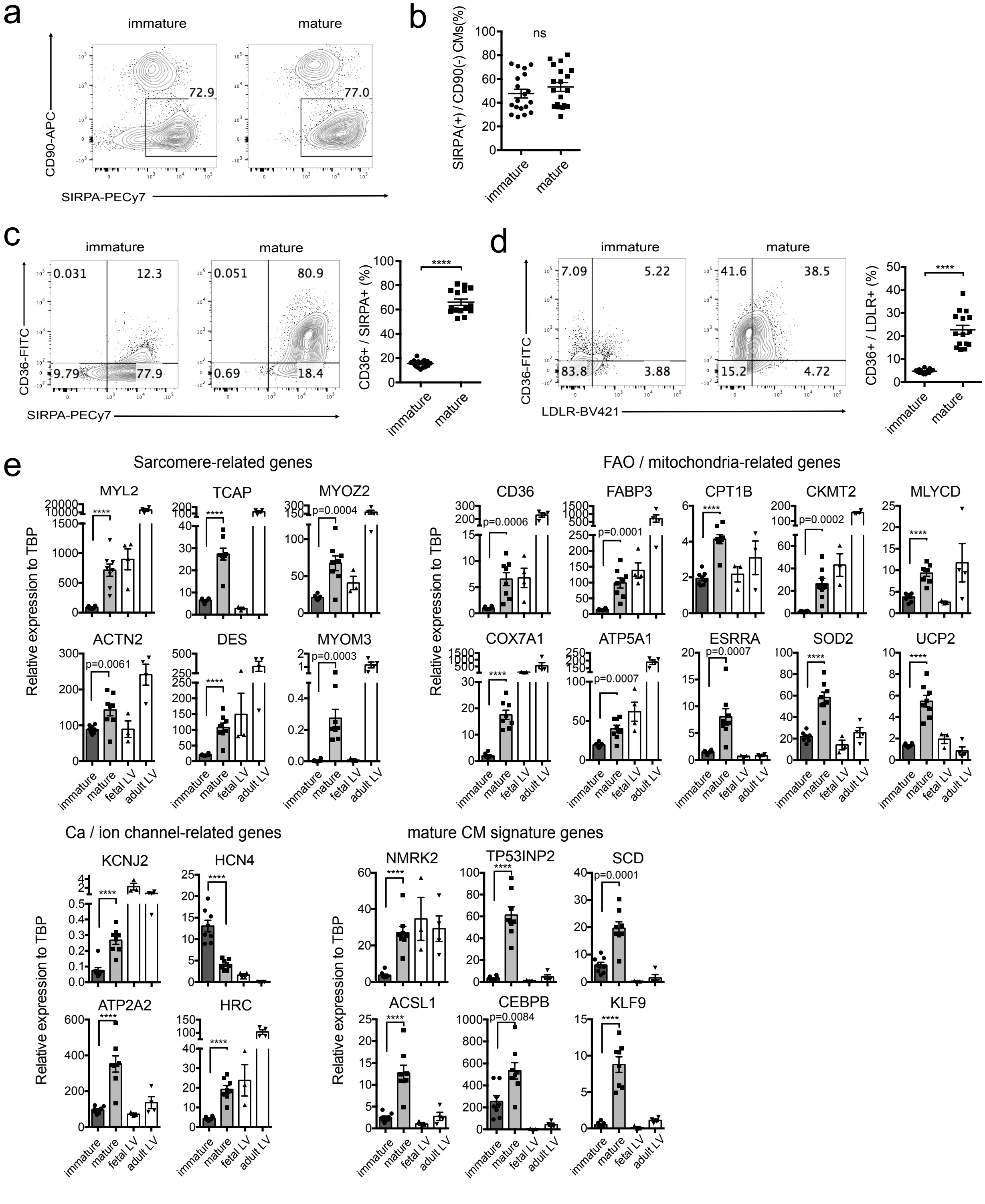

Supplementary Figure 7. Maturation of ventricular cardiomyocytes from the ESI-17 hESC line. (a) Representative flow cytometric analyses of CD90 and SIRPA in the immature and mature day 32 cardiomyocytes. (b) Comparison of the percentage of SIRPA(+) and CD90(-)

cardiomyocytes in the immature and mature cardiomyocytes (N=19). (c) Left: Representative flow cytometric analyses of CD36 and SIRPA in the immature and mature cardiomyocytes. Right: Quantification of CD36(+) and SIRPA(+) cardiomyocytes in the immature and mature cardiomyocytes (N=15). (d) Left: Representative flow cytometric analyses of CD36 and LDLR in the immature and mature cardiomyocytes. Right: Quantification of CD36(+) and LDLR(+) cardiomyocytes in the immature and mature cardiomyocytes (N=15). (e) RT-qPCR expression analyses of sarcomere, FAO, mitochondria, Ca2+, ion channel-related genes and mature cardiomyocyte-signature genes in the indicated populations (N=8). RT-qPCR was performed on isolated SIRPA(+)/CD90(-) cardiomyocytes as shown in (a). Fetal LV and adult LV tissues were included as a reference for *in vivo* expression. All statistical analyses were performed by two-sided unpaired t-test (****p<0.0001). Data are presented as mean values +/- SEM. Fetal LV: Fetal left ventricular tissue (N=3-4), Adult LV: Adult left ventricular tissue (N=3-4).

Supplementary Figure 8

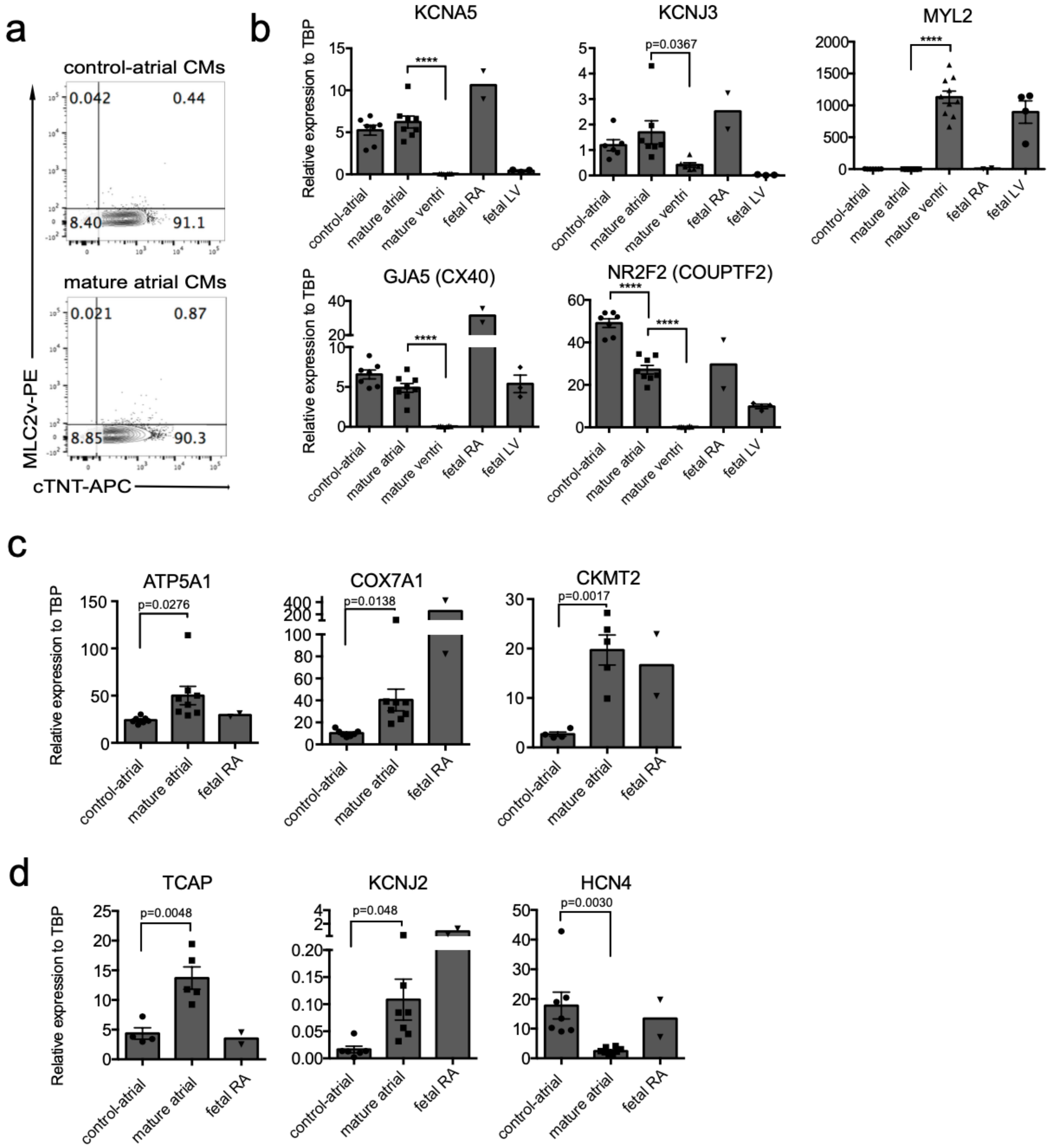

Supplementary Figure 8. Maturation of atrial cardiomyocytes. (a) Representative flow cytometric analyses of MLC2v and cTNT in control and mature day 32 atrial cardiomyocytes. (b) RT-qPCR expression analyses of atrial specific genes and *MYL2* in the indicated populations (N=7 biologically independent experiments for control atrial, N=8 for mature atrial, and N=6 for mature ventricular for atrial specific genes. N=7 for control atrial, N=8 for mature atrial, and N=10 for mature ventricular for MYL2). (c) RT-qPCR expression analyses of mitochondrial genes (N=7 for

control, N=8 for mature for ATP5A1 and COX7A1, N=4 for control, N=5 for mature for CKMT2) and (d) *TCAP, KCNJ2*, and *HCN4* in the indicated populations (N=7 for control, N=8 for mature for KCNJ2 and HCN4. N=4 for control, N=5 for mature for TCAP). Fetal LV and RA tissues were included as a reference for *in vivo* expression in RT-qPCR analyses. Statistical analyses were performed by two-sided unpaired t-test (c, d) or one-way ANOVA with Tukey's multiple comparisons (b) (****p<0.0001). Data are presented as mean values +/- SEM. Fetal LV: Fetal left ventricular tissue (N=3-4), Fetal RA: Fetal right atrial tissue (N=2).

Supplementary Figure 9

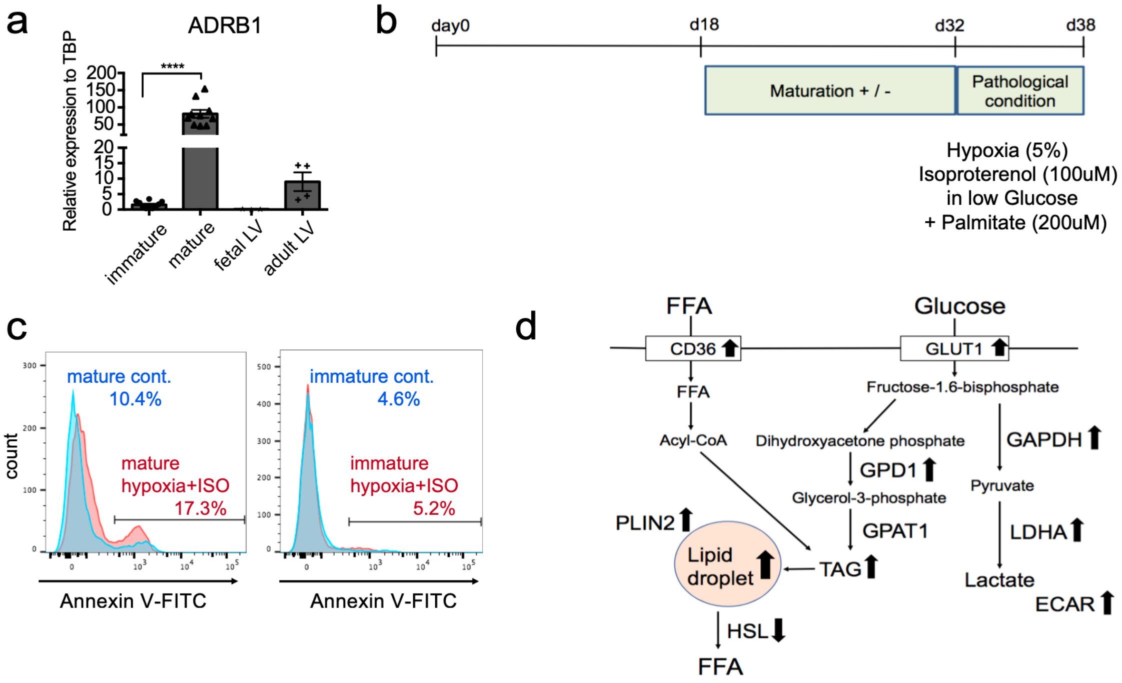

Supplementary Figure 9. Modelling pathological adaptation using mature compact cardiomyocytes. (a) RT-qPCR expression analyses of *ADRB1* in immature and mature day 32 ventricular cardiomyocytes (N=10). Fetal LV and adult LV tissues were included as a reference for *in vivo* expression. Statistical analyses were performed by two-sided unpaired t-test (****p<0.0001). (b) Schematic of the protocol used for modeling pathological stimuli in vitro. (c) Representative flow cytometric analyses of Annexin V in the non-stimulated and hypoxia+ISO stimulated immature and mature ventricular cardiomyocytes. (d) Summary of the pathways activated in hypoxia+ISO stimulated mature cells. Black arrows indicate genes and parameters activated (or downregulated) in the stimulated mature cells. Data are presented as mean values +/- SEM. Fetal LV: Fetal left ventricular tissue (N=3), Adult LV; adult left ventricular tissue (N=4).

Supplementary Figure 10

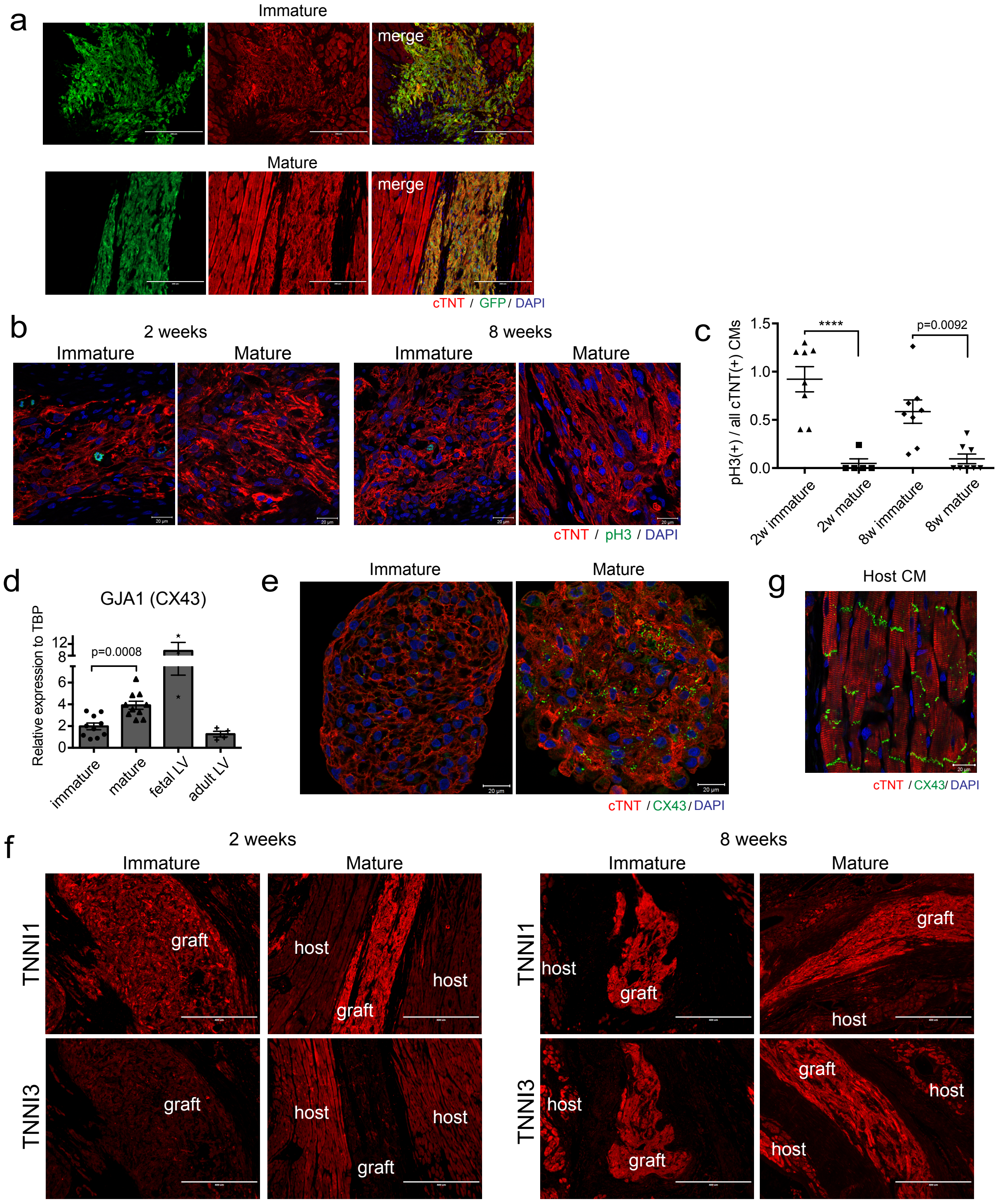

Supplementary Figure 10. Engraftment of mature and immature cardiomyocytes in infarcted rat hearts. (a) Representative immunostaining of cells in grafts generated from mature

and immature cells at 2 weeks post transplantation. Scale bar:200um. Similar staining patterns were observed in all the transplanted animals (N=8 recipients of 2-week immature cells, N=5 recipients of 2-week mature cells). (b) Representative immunostaining showing $pH3^+$ cells in cardiomyocyte grafts generated from immature and mature cells at 2 and 8 weeks post transplantation. Scale bar:20um. (c) Quantification of the percentage of $pH3^+$ cardiomyocytes in grafts derived from immature and mature cells (N=8 recipients of 2-week immature cells, 5 recipients of 2-week mature cells, 8 recipients of 8-week immature cells, and 8 recipients of 8-week mature cells). Statistical analyses were performed by one-way ANOVA with Tukey's multiple comparisons (****p<0.0001). (d) RT-qPCR expression analyses of *GJA1* (CX43) in immature and mature day 32 EBs (N=10). Fetal LV and adult LV tissues were included as a reference for *in vivo* expression. Statistical analyses were performed by two-sided unpaired t-test. (e) Representative immunostaining showing CX43 expression in mature and immature day 32 EBs. Scale bar:20um. Three independent experiments were repeated with similar results. (f) Representative immunostaining showing the expression pattern of TNNI1/3 in cardiomyocyte grafts generated from immature and mature cells at 2 and 8 weeks post transplantation. Scale bar:400um. Similar staining patterns were observed in all the transplanted animals (N=8 recipients of 2-week immature cells, 5 recipients of 2-week mature cells, 8 recipients of 8-week immature cells, and 8 recipients of 8-week mature cells). (g) Representative immunostaining showing CX43 expression patterns in host rat cardiomyocytes. Similar staining patterns were observed in all the transplanted animals (N=8 recipients of 2-week immature cells, 6 recipients of 2-week mature cells, 8 recipients of 8-week immature cells, and 8 recipients of 8-week mature cells). Data are presented as mean values +/- SEM. Fetal LV: Fetal left ventricular tissue (N=3), Adult LV; adult left ventricular tissue (N=4).

Supplementary Figure 11

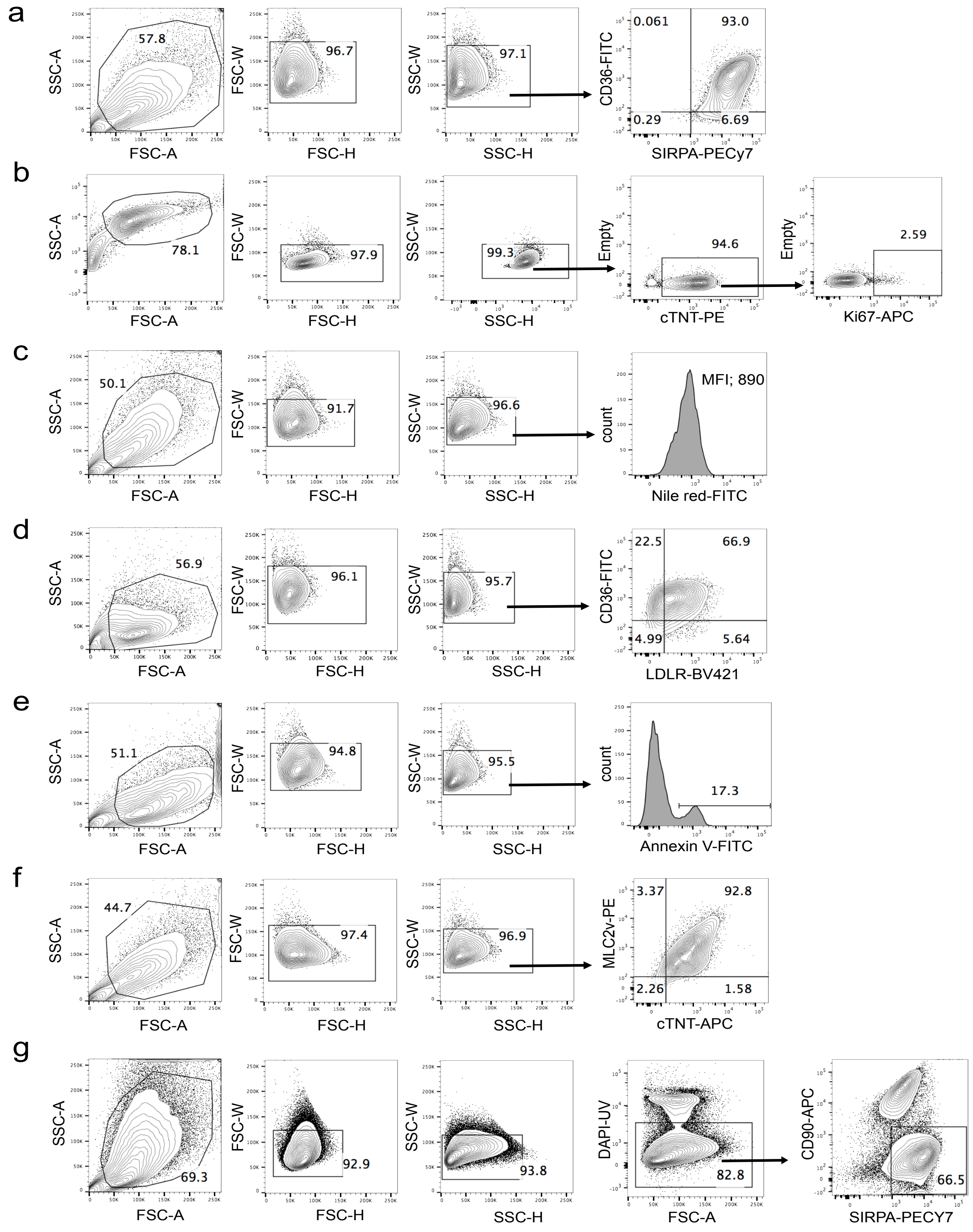

Supplementary Figure 11. Gating strategy used for flow cytometric analyses. (a) Gating strategy to determine the percentage of CD36 and SIRPA-positive cells shown in Figure 2, 7, and Supplementary Figure 2, 7. (b) Gating strategy to determine the percentage of Ki67-positive

cells among cTNT-positive cardiomyocytes shown in Fig 1, and Supplementary Figure 1, 2, 4. (c) Gating strategy for Nile red staining shown in Figure 2, 3, 6, 8. (d) Gating strategy to determine the percentage of CD36 and LDLR-positive cells shown in Figure 6 and Supplementary Figures 6, 7. (e) Gating strategy for Annexin V staining shown in Figure 8 and Supplementary Figure 9. (f) Gating strategy to determine the percentage of cTNT and MLC2v-positive cells shown in Supplementary Figure 5, 8. (g) Gating strategy to determine the percentage of SIRPA-positive and CD90-negative cells shown in Supplementary Figure 7.

Supplementary Table 1: Quality Control data of scRNAseq in day20 ventricular cells and day32 mature and immature cells.

Sample	Estimated Number of Cells	Mean Reads per Cell	Mean Genes per Cell	Number of Reads	Reads Mapped confidently to Genome
Day20 ventricular	3,620	57,554	3,438	208,346,707	90.80%
Day32 mature	3,285	20,721	2,167	68,070,706	91.20%
Day32 Immature	2,741	27,798	2,646	76,194,368	89.30%

Supplementary Table 2: Differentially expressed genes (top 24 genes) in each cluster among all cells

rank	cluster_0	cluster_1	cluster_2	cluster_3	cluster_4
1	NMRK2	MDK	TMSB4X	ACTA2	SERPINA1
2	CKMT2	NREP	S100A11	BTG1	TTR
3	MYH6	MT1E	SPARC	MYLK	TM4SF4
4	COX6A2	FXYD6	LGALS1	VSNL1	CLU
5	MASP1	NKX2-5	COL3A1	MYH11	ACTG1
6	HRC	H3F3B	ACTG1	NES	GC
7	CMYA5	MT1G	COL1A2	NR2F2	S100A10
8	GOT1	MT2A	FN1	AKAP12	BEX1
9	NDUFB3	BEX4	COL6A2	BMP2	TMSB4X
10	ACSL1	PTP4A3	TIMP1	DES	KRT19
11	ASAH1	TSC22D1	EVA1B	NR2F1	S100A11
12	IGFBP7	ENO3	VIM	RRAD	CYBA
13	MYBPC3	BANCR	TPM4	CXCL12	MARCKSL1
14	TP53INP2	CLU	TAGLN2	A2M	SPINT2
15	NEBL	C12orf75	COL1A1	SFRP1	DLK1
16	NPPB	FGF18	BGN	TAGLN	TM7SF2
17	SDHB	CNN1	TMSB10	MYH6	ZFAS1
18	DES	SLC30A1	MARCKSL1	CPNE5	TXN
19	CKB	MYL6B	C11orf96	IGFBP5	RBP1
20	ANKRD9	IFI27L2	SH3BGRL3	ID3	CLIC1
21	CEL	PLCG2	IFITM3	PDLIM3	HMGN1
22	SDHA	TGFB1I1	MARCKS	ID2	TMSB10
23	ACTN2	MAGED2	GNG11	AEBP1	SH3BGRL3
24	CD36	MEIS2	C7	TGFB1I1	SOX4

Supplementary Table 3: Differentially expressed genes (top 24 genes) in each cluster by clustering analyses among mature CMs

rank	cluster_A	cluster_B	cluster_C	cluster_D
1	MYH7	ASNS	STMN1	TMSB4X
2	COL11A2	PHGDH	TYMS	SPARC
3	LINC01088	SARS	PTTG1	COL3A1
4	ASB2	RNF187	HMGN2	FN1
5	FGF12	TRIB3	CDKN3	LGALS1
6	HEPH	ARG2	ANLN	COL1A1
7	NREP	GARS	CENPM	COL1A2
8	ALDOC	BEX2	SMC4	S100A11
9	RYR2	SHMT2	BIRC5	COL6A2
10	MTRNR2L8	NUPR1	RRM2	TPM4
11	GUCY1A1	ATF5	FOXM1	MARCKSL1
12	PYGM	EIF3E	TK1	FLNA
13	LGALS3BP	ZFAS1	UBE2T	C7
14	LDLR	TCEA1	KPNA2	POSTN
15	FNDC5	SESN2	CENPF	C11orf96
16	PHACTR1	ATF4	ACTA2	MGP
17	DMPK	MTHFD2	NUSAP1	EVA1B
18	NAV1	HERPUD1	DHFR	TAGLN2
19	PCDH7	DDIT3	CENPW	RGS5
20	HSPB6	SLC3A2	MKI67	BGN
21	CEL	WARS	RPL39L	TIMP1
22	ROGDI	XBP1	HMGB2	MARCKS
23	CD36	IARS	PCLAF	SH3BGRL3
24	SRL	PSAT1	TPX2	COLEC11

Supplementary Table 4: Genes whose expression is not impacted by the knockdown of ESRRA expression.

Sarcomere gene	Ca^{2+}, ion channel, gap junction gene	FAO, metabolic gene
MYH7	ATP2A2	CD36
MYH6	HCN4	CPT1B
MYL7	GJA1 (CX43)	CKMT2
ACTN2	HRC	MLYCD
MYLK3		ACC2
MYOZ2		COX7A1
DES		ATP5A1
MYOM3		ESRRG
MYBPC3		PPARA
		COM6A2
		ATP1A3

Supplementary Table 5: Primer lists

Gene	Forward 5'-3'	Reverse 5'-3'
TBP	TGAGTTGCTCATACCGTGCTGCTA	CCCTCAAACCAACTTGTCAACAGC
HEY2	GAGTGAGAGAGTCGTGTTTC	ACTTCTGTCCCTTTCCTTTC
MYCN	CCAGCAGATGCCACATAAG	CCTCTCATTACCCAGGATGTA
ANF	GGGTCTCTGCTGCATTTGTGTCAT	AGAGGCGAGGAAGTCACCATCAAA
BMP10	GCCCATCTCCATCCTCTATTT	AAGCCATAGGACTCTTCTTCTATC
CD36	GGTGATGAGAAGGCAAACA	ACATCACCACACCAACAC
FABP3	CACTCGCACTTATGAGAAAGA	AGGAAGAAATGAGGCAATGT
CPT1B	TGGAAACCACATCCGCAAAG	AAGGGCAGCTGGCATTTC
MLYCD	AACAGCACCTCCTACCT	TCAGAGCTTGCTGTTCTTT
ACC2	GTTCACCTGCTGTCTACCAT	GGTCTGGGTGGTTCCTTT
ATP5A1	GGCTGATGGAAAGATCTCAG	GATGTGAATCCACAGGAGTTTA
COX7A1	CTACAGCTTGTACTCCCTTG	CAGAGGCCAGCGTTTATT
CKMT2	GGAGAGAGGCCAAGATATTAAG	CGTACCAGCAGACAGATTATT
SOD2	CACGCTTACTACCTTCAGTATAAA	TTCTCCCAGTTGATTACATTCC
UCP2	CTATGAGCAGCTGAAACGAG	AGAGACAAAGCCAGAGGT
GLUT1	CCTGGTTCTGTTCTTCATCTT	CGGGTGTCTTGTCACTTT
GAPDH	AGGTGGTCTCCTCTGACTTCAACA	GACAAAGTGGTCGTTGAGGGCAAT
LDHA	TCCTTAGTGTTCCTTGCATTT	AAGTGTATCTGCACTCTTCTTC
GPAT1	CCTCAATGCAACCGACAA	TACCTACAAGGAACTCATCTCA
GPD1	CCTTGTTCATGGCTGTGT	CACTCACATATGTTCTGGATGA
PLIN2	AATTCAGGATGCTCAGGATAAG	ACAGTGGGACTCATCAGTAT
HSL	GATTCCCTCAAGAACCTGAC	GCATCCTCAGGTGGTAATAAG
CASP9	TCGCTAATGCTGTTTCGG	CCCTGGCCTTATGATGTTT
TNNT2	TTCACCAAAGATCTGCTCCTCGCT	TTATTACTGGTGTGGAGTGGGTGT
IRX4	TTGGACTCCTGGGAACATGGACAA	ATGCTTCAGGGTATCTGGCCTCTT
KCNA5	AGTGTAACGTCAAGGCCAAGAGCA	TCACAAATCTGTTTCCCGGCTGGT
KCNJ3	TCATCAAGATGTCCCAGCCCAAGA	CACCCGGAACATAAGCGTGAGTTT
COUPTF2	TGATGTAGCCCATGTGGAAAG	GCTGCCGGACAGTAACATATC
CX40	AATCTTCCTGACCACCCTGCATGT	CAGCCACAGCCAGCATAAAGACAA
MYL2	TGTCCCTACCTTGTCTGTTAGCCA	ATTGGAACATGGCCTCTGGATGGA
TCAP	CCATGGCTGCTTTGTAGTT	CCAACTCTCCACCCTTAGT
KCNJ2	CTTTGTAGTGCCAGAGACTTAG	CACTGTCGTCTTCCTCTTTG
HCN4	TCTTCCTCATTGTGGAGACACGCA	TGAGGATCTTCGTGAAGCGGACAA
ADRB1	CCTCTTCGTCTTCTTCAACTG	AGAGCAGTCCCTGGAAG
CX43	GATGACCAGTCGGCCTTTCGTTGT	AGAAGCGCACATGAGAGATTGGGA
COX6A2	CCACACTCTGTTCCACAATAG	TCGAAGCTTCACACCTTTATT

MYOZ2	CCCAGGTTTATGTCCTTTGT	ATCTGTAGGTTCGGTTGTTATC
ESRRA	ACTTAGTCCTGGATGAAGAGG	GCCAAGGCCTTTAGTAGAAC
ACSL1	CATGAGCTGTTCCGGTATTT	GAAGCCCATAAGCGTGTT
IRX3	TCTGCCTTTGTGTGTGTG	GTGGCAGCAGCTCATTTA
TBX20	GGGTATCATCTTCTTCCAGTTT	CATTCCCAGTCGGCTATATG
NPPB	TCCTGCTCTTCTTGCATCTGGCTT	TGTAACCCGGACGTTTCCAAGT
HAS2	TCCAGCTAGTAGGTCTCATAAA	CATCTTGGCGGGAAGTAAA
FZD1	CCTCTTCGTGTACCTGTTTATC	TGAGCTTCTCCAGCTTCT
HRC	GGCAGACATGCTGGAAA	TAAATATAGCTCGTGGAAAGGG
ACTN2	ATTACGCTGCGTTCTCTTC	CTGATGGGATGAGTGATTACAG
DES	GTGAGGCCACACAGCAG	TTCAGGGAGCAGTGAGGA
MYOM3	GTTGCTCTCGTAACCATGAC	AGCCTGTGAACCTCCAT
NMRK2	CAGCCCGCAGAAGTTTG	TGTAGCTGTAGAGCAGGAAG
TP53INP2	CTCTGGAGATTGGTTCACCTT	CTGGGAGGCAGCGATTT
SCD	CAACTACCACCACTCCTTTC	GAGACTTTCTTCCGGTCATAG
CEBPB	CAAGAGCAAGGCCAAGAA	GAGCTCCAGGACCTTGT
KLF9	AAAGTTCTCCCGCTCAGA	GAGGTGGTCACTCCTCAT
